# An experimental study of the effects of electronic cigarette warnings on young adult nonsmokers’ perceptions and behavioral intentions

**DOI:** 10.1186/s12971-016-0083-x

**Published:** 2016-05-26

**Authors:** Darren Mays, Clayton Smith, Andrea C. Johnson, Kenneth P. Tercyak, Raymond S. Niaura

**Affiliations:** Department of Oncology, Georgetown University Medical Center, Lombardi Comprehensive Cancer Center, 3300 Whitehaven St NW Suite 4100, Washington DC, 20007 USA; Schroeder Institute for Tobacco Research & Policy Studies, Truth Initiative, 900 G Street NW, Washington DC, 20001 USA

**Keywords:** Electronic cigarettes, Warning labels, Young adults, Non-smokers

## Abstract

**Background:**

Electronic cigarette (“e-cigarette”) manufacturers use warning labels on their advertising that vary widely in content and the U.S. Food and Drug Administration has issued a warning label requirement for e-cigarettes. There is limited data on the effects of these warnings on e-cigarette perceptions and other potential predictors of future tobacco use behavior in populations of interest to inform future regulatory requirements. This study examined the effects of e-cigarette warnings on perceptions of e-cigarettes and cigarettes and other cognitive precursors to tobacco use among young adult non-smokers.

**Methods:**

Non-smoking young adults ages 18 to 30 years (*n* = 436) were recruited through an internet-based crowdsourcing platform for an online experiment. Participants completed pre-exposure measures of demographics, tobacco use, and other relevant constructs and were randomized to view 1 of 9 e-cigarette stimuli in a 3 (Ad/Warning condition: Ad Only, Ad with Warning, Warning Only) x 3 (E-cigarette brand: Blu, MarkTen, Vuse) design. After viewing e-cigarette stimuli, participants reported perceptions of e-cigarettes and behavioral intentions to use e-cigarettes. Participants in the Ad Only and Ad with Warning conditions also completed a heat-mapping task assessing aspects of the ads that captured their attention. Then, participants were randomized to view cigarette ads from 1 of 3 major cigarette brands and reported perceptions of cigarettes and intentions to smoke cigarettes.

**Results:**

Participants in the Warning Only condition reported significantly greater perceived harm and addictiveness of e-cigarettes and thoughts about not using e-cigarettes than the Ad Only and Ad with Warning conditions (*p*’s < .05). The Ad Only and Ad with Warning conditions did not differ on these outcomes. Participants in the Warning Only condition also reported the harms of e-cigarettes were closer to those of cigarettes than the Ad Only condition (*p* < .05), but neither differed from the Ad with Warning condition. Visual inspection of heat-mapping task data indicate warnings drew few participants’ attention. There were no significant differences across study conditions on perceptions of cigarettes or intentions to smoke.

**Conclusions:**

Text-based warning messages influenced young non-smokers’ perceptions in a way that may dissuade e-cigarette use, but warnings appearing on advertisements had little impact.

**Electronic supplementary material:**

The online version of this article (doi:10.1186/s12971-016-0083-x) contains supplementary material, which is available to authorized users.

## Background

Population data indicate electronic cigarette (e-cigarette) use has increased in recent years in the U.S [1, 2]. From 2010 to 2013, lifetime e-cigarette use among adults increased from 3.3 to 8.5 % [3]. Additionally, the available research indicates the prevalence of e-cigarette use is higher among young adults than all other adult age groups [3–5]. An analysis of U.S. population data collected in 2014 indicated 16.5 % of young adults ages 18 to 24 had ever tried e-cigarettes but did not currently use them, and 5.2 % were current (i.e., some days or every day) e-cigarette users [6]. E-cigarette advertising has also become more prominent in recent years with ads appearing online, in print media, and on television and radio [7]. E-cigarette ads often make implicit or explicit health-related claims (e.g., e-cigarettes are a safer alternative to cigarettes) and deploy strategies (e.g., celebrity endorsers) to enhance the appeal of the products [8]. Thus, advertising is likely a major factor contributing to e-cigarette use [8–10].

Experimental data show that e-cigarette ads affect perceptions of e-cigarettes and combustible cigarettes. E-cigarette ads produce urges to smoke among cigarette smokers and reduce former smokers’ self-efficacy and intentions to remain abstinent [11]. Adult smokers are highly receptive to e-cigarette ads, and ads produce intentions to try e-cigarettes and elicit thoughts about smoking and quitting cigarettes [10]. E-cigarette ads have also been shown to prompt stronger intentions to use e-cigarettes in adolescents [12].

According to recent data young adults’ exposure to e-cigarette advertising is increasing [13] and research indicates young adults perceive e-cigarettes to be convenient, modern, and less harmful than cigarettes, themes that commonly appear in e-cigarette ads [14]. Although the prevalence of e-cigarette use among U.S. adult non-smokers remains low [6], a recent analysis of population data estimated that one quarter of U.S. young adults who do not use e-cigarettes are open to using them in the future, and more than one third of young adults who are open to using e-cigarettes are non-cigarette smokers [15]. Recent longitudinal data also indicate young adulthood is a period when e-cigarette initiation occurs [16].

In May 2016, the Food and Drug Administration (FDA) issued a “deeming” rule bringing e-cigarettes under the FDA’s regulatory authority [17, 18]. The rule includes a warning label requirement for e-cigarette packaging and advertising reading: “WARNING: This product contains nicotine. Nicotine is an addictive chemical.” The warning requirements will go into effect 24 months after publication of the final rule. Before this final rule was issued, several e-cigarette manufacturers have included warnings on their ads even though they were not required, which range from lengthy messages about potential harms to brief statements about addiction [19].

Warning labels communicating the potential risks of tobacco products are one component of a comprehensive approach to tobacco control. However, unlike research on cigarette warning labels, there is limited evidence on whether e-cigarette warning labels, including warnings currently used by manufacturers and the warning required by FDA, affect young adults’ perceptions of e-cigarettes and intentions to use them. One recent experimental study conducted among U.S. young adults showed that warnings on e-cigarette television advertisements reduced cravings for cigarettes and e-cigarettes among users of these products and reduced intentions to purchase e-cigarettes [20]. The study also showed that warnings conveying that some e-cigarettes are produced by cigarette companies increased perceptions that e-cigarettes are harmful, but reduced perceptions that e-cigarettes are addictive [20]. However, the study did not investigate the potential effects of the warning proposed by FDA in 2014, the warning now required in the final rule, or warnings used by manufacturers. Another recent experimental study showed that adult cigarette smokers and dual users of cigarettes and e-cigarettes who were exposed to a warning label currently used by one e-cigarette manufacturer were more likely to perceive the product to be harmful, however the study did not examine potential effects of the warning among non-smokers [21]. Additionally, given the evidence that young adult e-cigarette use is associated with use of combustible cigarettes, warnings on e-cigarette ads may affect perceptions of cigarettes and intentions to smoke [20], but this has not been investigated. The objective of this study was to examine the effects of warning labels on e-cigarette ads on young adult non-smokers’ perceptions of e-cigarettes and behavioral intentions, and to investigate their effects on perceptions of combustible cigarettes and intentions to smoke.

## Methods

### Setting and sample

Participants were recruited through Amazon Mechanical Turk (AMT) [22], a crowdsourcing data collection platform used in similar studies of tobacco use [23–25] and other risk behaviors [26, 27]. After a brief description of the study, individuals residing in the U.S. who were interested in participating reviewed a complete study description with a link to a consent form and eligibility screener. Non-smokers ages 18 to 30 were eligible to participate. We focused on young adult non-smokers because this is a period when tobacco use initiation and transitions often occur, e-cigarette use is prevalent among young adults, and because young adults are targeted by tobacco industry advertising [28–30]. Age and cigarette smoking were assessed at screening, with those outside the target age range and those reporting smoking ≥ 100 lifetime cigarettes and now smoking every day or some days [5] excluded. Eligible, consenting individuals proceeded to the online experiment. Participants completing all procedures were given a monetary credit through AMT.

### Procedures

Study procedures occurred at a single time point through a series of steps. Participants reviewed a brief description of e-cigarettes to orient them to the study topic [5] and completed pre-exposure measures. Then, using an algorithm in the online survey participants were randomized to view 1 of 9 e-cigarette ad stimuli in a 3 (Ad/Warning condition: Ad Only, Ad with Warning, Warning Only) by 3 (Brand: Blu, MarkTen, Vuse) factorial experiment. Participants randomized to the Ad Only condition (*n* = 144, 33.0 %) viewed an ad without a warning. Participants randomized to the Ad with Warning condition (*n* = 147, 33.7 %) viewed an ad with a warning. Those randomized to the Warning Only (*n* = 145, 33.3 %) condition viewed a warning label alone, independent of any ad. Participants viewed an e-cigarette ad with or without a warning for the brand to which they were randomized (Blu [*n* = 138, 31.6 %], MarkTen [*n* = 147, 33.7 %], or Vuse [*n* = 151, 34.6 %]), or a warning label only. The warning in the Blu condition was the warning in FDA’s 2014 proposed deeming rule, which is similar in contents to that of the final rule issued in 2016. The warning in the MarkTen condition was a lengthy warning about potential health risks used by this manufacturer on its ads at the time of the study. The warning in the Vuse condition was also a warning used by this manufacturer, a brief message about potential addiction. Print ads for each brand were obtained from a publicly available tobacco advertising database [31]. Ads were sized to identical dimensions and edited to remove minor differences for consistency across conditions (e.g., web site addresses). Warnings were displayed with consistent size, font, and placement across conditions. E-cigarette ad stimuli are available in Additional file 1. Participants viewed the e-cigarette ad stimuli for as long as they wished, and then completed e-cigarette outcome measures. While viewing the ads, participants in the Ad Only and Ad with Warning conditions also completed a heat map task where they were asked to identify up to 5 areas in the e-cigarette ads that attracted their attention by pointing their cursor and clicking on the areas of the e-cigarette ad image [32].

After completing e-cigarette outcome measures, participants were randomized to view a Camel (*n* = 150, 34.4 %), Marlboro (*n* = 140, 32.1 %), or Newport (*n* = 146, 33.5 %) cigarette advertisement. We chose these brands because they are among the most widely advertised and top-selling cigarette brands in the U.S. We randomly assigned ads for three different cigarette brands to account for any potential impact of brand familiarity or preference within the design. Similar to e-cigarette stimuli, cigarette ads were sized and presented consistently across conditions. After viewing cigarettes ads, participants completed cigarette outcome measures.

This study was approved as exempt by the Georgetown University Institutional Review Board. All participants provided informed consent.

### Measures

#### Pre-exposure measures

Prior to the experimental exposure, demographics assessed included age, gender, race/ethnicity, household income, employment, and current college/university student status. Based on epidemiological items administered at screening [5], we created a variable categorizing participants as never smoking, trying cigarettes (i.e., tried smoking cigarettes but have not smoked 100 lifetime cigarettes), or experimenting (i.e., smoked 100 or more lifetime cigarettes but do not currently smoke on all or some days). A single item also measured whether participants had ever tried e-cigarettes (yes/no) [5]. Another item measured exposure to e-cigarette advertising in magazines, on television, or convenience stores based on a five point scale [33, 34]. Four items also assessed past 30 day use of smokeless tobacco; cigars; little cigars and cigarillos; and waterpipe (hookah) tobacco use [5]. These were combined into a binary variable indicating use of any of these products in the past 30 days to characterize the sample.

#### Outcome measures

Outcome measures were based on a tobacco warning label science theoretical framework emphasizing consumer perceptions and behavioral intentions as cognitive antecedents to future behavior [35]. After exposure to the e-cigarette ad stimuli, perceived harms and addictiveness of e-cigarettes were measured with two items asking how harmful/addictive e-cigarettes are on a four point scale (1 = not at all, 2 = slightly, 3 = somewhat, 4 = very harmful) [23, 36]. Perceived harms and addictiveness of e-cigarettes relative to cigarettes were measured with two items asking participants whether e-cigarettes are less or more harmful/addictive than cigarettes [23, 36]. Response options were on five point scale (1 = much less, 2 = less, 3 = about the same, 4 = more, 5 = much more). Two items assessed thoughts about not using e-cigarettes on a 7-point scale [37]. Responses were averaged to create a summary score with higher values indicating more thoughts about not using e-cigarettes (Cronbach’s α = 0.89). Behavioral intentions to use e-cigarettes were measured using an item asking how likely it is participants would use an e-cigarette in the next year based on a four point response scale (1 = definitely will not, 4 = definitely will) [5, 38, 39].

After exposure to the cigarette ads, perceived harms and addictiveness of cigarettes were measured with two items with the same response scale as the e-cigarette items [23, 36]. Perceived harms and addictiveness of cigarettes relative to e-cigarettes were also measured with two items asking participants to indicate how harmful/addictive cigarettes were to a person’s health relative to e-cigarettes with response options similar to the e-cigarette items [23, 36]. Intentions to use cigarettes were measured with an item similar to the e-cigarettes measure, with response options on a four point scale (1 = definitely will not, 4 = definitely will) [5, 38, 39].

### Statistical analyses

Bivariate analyses confirmed no participant characteristics differed (*p* < .05) by experimental conditions. However, all multivariable analyses included covariates for prior e-cigarette use and e-cigarette advertising exposure to account for potential effects on study outcomes. Analysis of Covariance (ANCOVA) was used to examine differences in e-cigarette outcomes based on main effects for Ad/Warning condition, e-cigarette brand, and their interaction, adjusting for covariates noted above. Least-square means were examined for significant main and interaction effects, accounting for multiple comparisons using Tukey’s adjustment. For cigarette outcomes, ANCOVA was used to examine differences based on main effects for Ad/Warning condition, e-cigarette brand, cigarette brand, and all two-way interactions. E-cigarette use, e-cigarette advertising exposure, and prior cigarette smoking were included as covariates.

## Results

### Sample

In total, 1,018 individuals responded to study eligibility screening questions, 436 of whom (42.8 %) were eligible, randomized, and completed all study procedures. Among those who were ineligible (*n* = 582), 160 (27.5 %) were nonsmokers older than age 30, 177 (30.4 %) were smokers ages 18–30, and 245 (42.1 %) were smokers older than age 30. Participants averaged 25.0 years of age (*SD* 3.2 years), 55 % were male, 81 % were white, and 93 % completed at least some college education (Table 1).

**Table 1:** Sample Characteristics (*n* = 436)

	Mean (SD) or % (n)
Age (M, SD)	25.0 (3.2)
Gender (%, n)	
Male	55.3 % (241)
Female	44.7 % (195)
Race (%, n)	
Black/African American	6.0 % (26)
White	81.4 % (354)
Other	12.7 % (55)
Hispanic ethnicity (%, n)	10.6 % (46)
College/University student (%, n)	
Current student	36.6 % (159)
Non-student	63.5 % (276)
Education (%, n)	
< High school	1.2 % (5)
High school grad or GED	6.4 % (28)
Some college	46.7 % (203)
College degree or higher	45.8 % (199)
Employment (%, n)	
Not employed	25.2 % (110)
Full time employed	52.1 % (227)
Part time employed	22.7 % (99)
Annual household income (%, n)	
< $20,000	21.6 % (94)
$20,001–$35,000	22.7 % (99)
$35,001–$50,000	21.3 % (93)
$50,001–$75,000	19.5 % (85)
> $75,000	13.3 % (58)
Prefer not to say	1.6 % (7)
Cigarette smoking (%, n)	
Never smoked	40.1 % (175)
Tried smoking	36.2 % (158)
Experimented	23.6 % (103)
Past 30 day use of other tobacco products (%, n)	
Yes	10.6 % (46)
No	89.5 % (390)
Ever used e-cigarettes (%, n)	
Yes	32.3 % (141)
No	67.7 % (295)
E-cigarette advertising exposure (M, SD, range 1 to 5)	3.1 (0.78)

### Perceptions of and behavioral intentions to use e-cigarettes

**Fig. 1:**
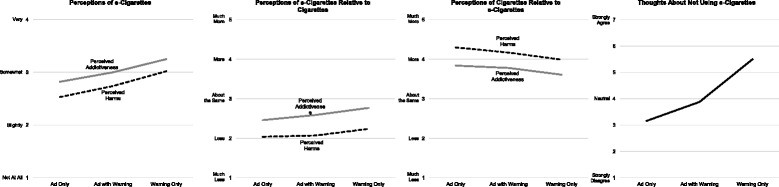
Least square means by Ad/Warning condition for e-cigarette and cigarette perceptions

There were significant main effects for Ad/Warning condition for perceived harms (*F* = 13.54, *η*^*2*^ = .060*, p* < .001) and addictiveness (*F* = 10.35, *η*^*2*^ = .047, *p* < .001) of e-cigarettes (Table 2). Participants in the Warning Only condition reported greater perceived harms of e-cigarettes (*M* 3.02, *SE* 0.07) than the Ad Only (*M 2.53*, *SE* 0.07*, p* < .001) and Ad with Warning (*M 2.74*, *SE* 0.07*, p* = .008) conditions (Fig. 1, Table 3). Participants in the Warning Only condition reported higher perceived addictiveness of e-cigarettes (*M* 3.25, *SE* 0.07) than the Ad Only (*M* 2.82, *SE* 0.07*, p* < .001) and Ad with Warning (*M* 3.00, *SE* 0.07*, p* = .02) conditions (Fig. 1, Table 3).

**Table 2:** Analysis of covariance (ANCOVA) results for perceptions of e-cigarettes, thoughts about not using e-cigarettes, and behavioral intentions to use e-cigarettes

	Perceived harms of e-cigarettes			Perceived addictiveness of e-cigarettes			Perceived harms of e-cigarettes relative to cigarettes			Perceived addictiveness of e-cigarettes relative to cigarettes			Thoughts about not using e-cigarettes			Intentions to use e-cigarettes		
	F	P	η^2^	F	P	η^2^	F	P	η^2^	F	P	η^2^	F	P	η^2^	F	P	η^2^
Main effects																		
Ad/Warning condition	13.54	<.001	.060	10.35	<.001	.047	3.12	.045	.015	7.11	<.001	.032	81.57	<.001	.278	1.80	.167	.008
E-cigarette brand	1.28	.279	.006	.20	.817	.001	.39	.677	.002	.95	.389	.004	1.50	.224	.007	.68	.505	.003
Interaction Effect																		
Ad/Warning condition x	.24	.918	.002	.83	.508	.008	.21	.933	.002	.59	.670	.006	2.29	.059	.021	.47	.758	.004
E-cigarette brand																		
Covariates																		
Ever used an e-cigarette	16.61	<.001	.038	12.32	<.001	.028	14.01	<.001	.032	9.87	.002	.023	15.09	<.001	.034	186.34	<.001	.305
E-cigarette advertising exposure	.58	.445	.001	1.36	.244	.003	2.18	.141	.005	1.19	.275	.003	.06	.810	<.001	.02	.891	<.001

**Table 3:** Comparisons of least square means for perceptions of e-cigarettes, thoughts about not using e-cigarettes, and behavioral intentions to use e-cigarettes based on main effects for ad/warning condition and brand

	Perceived harms of e-cigarettes		Perceived addictiveness of e-cigarettes		Perceived harms of e-cigarettes relative to cigarettes		Perceived addictiveness of e-cigarettes relative to cigarettes		Thoughts about not using e-cigarettes		Intentions to use e-cigarettes	
	M	SE	M	SE	M	SE	M	SE	M	SE	M	SE
Experimental condition												
Ad only^a^	2.53^C^	.07	2.82^C^	.07	2.04	.06	2.46^C^	.06	3.16^B,C^	.13	1.49	.05
Ad with warning^b^	2.74^C^	.07	3.00^C^	.07	2.07	.06	2.59	.06	3.88^A,C^	.13	1.58	.05
Warning only^c^	3.02^A,B^	.07	3.25^A,B^	.07	2.24	.06	2.77^A^	.06	5.50^A,B^	.13	1.44	.05
E-cigarette brand												
Blu^D^	2.75	.07	3.05	.07	2.08	.06	2.64	.06	4.24	.13	1.52	.06
MarkTen^E^	2.85	.07	3.04	.07	2.16	.06	2.54	.06	4.30	.13	1.45	.05
Vuse^F^	2.71	.07	2.99	.06	2.11	.06	2.65	.06	4.00	.13	1.53	.05

Covariates included ever e-cigarette use and e-cigarette advertising exposure. Means with different superscript letters within a column differ significantly at *p* < .05 in pair-wise comparisons after Tukey’s adjustment

For perceived harms of e-cigarettes relative to cigarettes, participants in the Warning Only condition (*M* 2.24, *SE* .06) reported e-cigarettes to be closer to cigarettes in harm than the Ad Only condition (*M* 2.04, *SE*.06, *p* = .055). There were no significant differences compared to the Ad with Warning condition (*M* 2.07, *SE* .06, Fig. 1, Table 3). Participants in the Warning Only condition reported e-cigarettes to be closer to cigarettes in addictiveness (*M* 2.77, *SE* .06) compared to the Ad Only (*M* 2.46, *SE* .06, *p* < .001) and Ad with Warning (*M* 2.59, *SE* .06, *p* = .075) conditions. The Ad Only and Ad with Warning conditions did not differ (*p* = .247, Fig. 1, Table 3).

For thoughts about not using e-cigarettes there was a significant main effect for Ad/Warning condition (*F* = 81.57, *η*^*2*^ = .278, *p* < .001, Table 2). Participants in the Warning Only condition reported significantly more thoughts about not using e-cigarettes (*M* 5.50*, SE* 0.13) than the Ad Only (*M* 3.16*, SE* 0.13, *p <* .001) and Ad with Warning (*M* 3.88*, SE* 0.13, *p <* .001) conditions. Thoughts about not using e-cigarettes were significantly greater in the Ad with Warning condition than the Ad Only condition (*p <* .001, Fig. 1, Table 3). For intentions to use e-cigarettes there were no statistically significant effects (Table 2). There were no statistically significant main effects for e-cigarette brand or interaction effects between Ad/Warning condition and e-cigarette brand for any e-cigarette perceptions or behavioral intentions outcomes (Table 2).

Results of the heat map task are shown in Additional file 2. Visual inspection of these data indicates that overall few participants indicated the warnings on the ads drew their attention compared with ads’ branded content.

### Perceptions of and behavioral intentions to use cigarettes

For perceived harms and addictiveness of cigarettes there were no significant effects (Table 4). For perceived harms of cigarettes relative to e-cigarettes, there was a significant main effect for Ad/Warning condition (*F* = 5.69, *η*^*2*^ = .027, *p* = .004, Table 4). Participants in the Warning Only condition reported cigarettes to be closer to e-cigarettes in perceived harm (*M* 3.99*, SE* 0.06) than the Ad Only condition (*M* 4.30*, SE* 0.06, *p =* .003, Fig. 1). For perceived addictiveness of cigarettes relative to e-cigarettes, the Ad/Warning condition main effect approached significance (*F* = 2.89, *η*^*2*^ = .014, *p* = .057, Table 4). There was a trend indicating that participants in the Warning Only condition reported cigarettes to be closer to e-cigarettes in perceived addictiveness (*M* 3.61*, SE* 0.07) than the Ad Only condition (*M* 3.84*, SE* 0.07, *p =* .055, Fig. 1, Table 5), however this difference was not statistically significant.

**Table 4:** Analysis of covariance (ANCOVA) results for perceptions of cigarettes and behavioral intentions to smoke

	Perceived harms of cigarettes			Perceived addictiveness of cigarettes			Perceived harms of cigarettes relative to e-cigarettes			Perceived addictiveness of cigarettes relative to e-cigarettes			Intentions to use cigarettes		
	F	P	η^2^	F	P	η^2^	F	P	η^2^	F	P	η^2^	F	P	η^2^
Main effects															
Ad/Warning condition	.82	.441	.004	.26	.774	.001	5.69	.004	.027	2.89	.057	.014	.85	.428	.003
E-cigarette brand	.35	.702	.002	1.32	.267	.006	1.30	.274	.006	.47	.623	.002	.80	.449	.003
Cigarette brand	.78	.461	.004	.04	.965	.002	.34	.713	.002	.56	.572	.003	3.40	.034	.014
Interaction effect															
Ad/Warning condition x	.35	.843	.003	.66	.624	.006	1.03	.392	.010	.43	.789	.004	2.21	.067	.018
E-cigarette brand															
Ad/Warning condition x	.89	.469	.009	.72	.580	.007	2.36	.053	.022	1.50	.202	.014	.42	.795	.003
Cigarette brand															
E-cigarette brand x	.81	.518	.008	.49	.744	.005	.75	.562	.007	.85	.491	.008	.95	.437	.008
Cigarette brand															
Covariates															
Ever used an E-cigarette	.04	.845	.001	.24	.622	<.001	15.90	<.001	.037	5.47	.020	.013	13.45	<.001	.027
E-cigarette Advertising exposure	.05	.829	.001	1.54	.215	.004	.66	.416	.002	.83	.364	.002	1.65	.199	.003
Cigarette smoking	.31	.576	<.001	.45	.502	.001	.04	.832	<.001	.23	.635	<.001	10.24	.002	.021

**Table 5:** Comparisons of least square means for perceptions of cigarettes and behavioral intentions to smoke cigarettes based on main effects for ad/warning condition, e-cigarette brand, and cigarette brand

	Perceived harms of cigarettes		Perceived addictiveness of cigarettes		Perceived harms of cigarettes relative to e-cigarettes		Perceived addictiveness of cigarettes relative to e-cigarettes		Intentions to smoke cigarettes	
	M	SE	M	SE	M	SE	M	SE	M	SE
Ad/Warning condition										
Ad only^a^	3.89	0.04	3.81	0.04	4.30^C^	0.06	3.84	0.07	1.30	0.04
Ad with warning^b^	3.89	0.03	3.84	0.04	4.17	0.06	3.78	0.07	1.22	0.04
Warning only^c^	3.84	0.04	3.80	0.04	3.99^A^	0.06	3.61	0.07	1.23	0.04
E-cigarette brand										
Blu^D^	3.87	0.04	3.77	0.04	4.24	0.07	3.78	0.07	1.26	0.04
MarkTen^E^	3.86	0.03	3.80	0.04	4.10	0.06	3.76	0.07	1.21	0.04
Vuse^F^	3.90	0.03	3.87	0.04	4.12	0.06	3.69	0.07	1.28	0.04
Cigarette brand										
Camel^G^	3.84	0.03	3.82	0.04	4.11	0.06	3.69	0.07	1.22^I^	0.04
Marlboro^H^	3.90	0.04	3.81	0.04	4.17	0.06	3.75	0.07	1.19	0.04
Newport^I^	3.87	0.03	3.81	0.04	4.18	0.06	3.79	0.07	1.34^G^	0.04

Note: Covariates included ever e-cigarette use, e-cigarette advertising exposure, and prior cigarette smoking. Means with different superscript letters within a column differ significantly at *p* < .05 in pair-wise comparisons after Tukey’s adjustment for multiple comparisons.

There was a significant main effect for cigarette brand for intentions to smoke cigarettes (*F* = 3.40, *η*^*2*^ = .014, *p* = 0.034, Table 4). Participants viewing Newport ads reported higher intentions to smoke (*M* 1.34*, SE* 0.04) compared to Marlboro ads (*M* 1.19*, SE* 0.04, *p* = 0.037, Table 5). There were no other significant effects for intentions to smoke (Table 4).

## Discussion

This study provides preliminary experimental evidence of the effects of warning labels on e-cigarette advertisements among young adult non-smokers. This investigation builds from other recent experiments [20, 21] by examining the warning statement such as that required by FDA’s deeming rule and warnings used by e-cigarette manufacturers within this population subgroup. Compared to ads with no warning labels and ads with warning labels, exposure to any of the three text-based e-cigarette warning labels tested independent of advertisements produced higher perceived harm and addictiveness of e-cigarettes and more thoughts about not using e-cigarettes. However, there were few differences in these outcomes between e-cigarette ads without warning labels and those that included warning labels. Self-report heat map data further supported the latter finding by indicating that branding elements on e-cigarette ads captured participants’ attention, but the warning labels did not. When examining perceived harm of cigarettes relative to e-cigarettes after participants viewed cigarette ads, e-cigarette warning labels viewed independent of ads produced perceptions that the harms of e-cigarettes are closer to those of cigarettes compared to e-cigarette ads without warning labels. There were no differences in any of the outcomes examined between FDA’s proposed warning and those used on ads by two major e-cigarette manufacturers, however this finding should be interpreted cautiously because the experimental design did not fully isolate these effects because different warning messages were not randomized across brands. Finally, we also observed no impact across experimental conditions on intentions to use e-cigarettes or intentions to smoke cigarettes.

FDA’s final deeming rule subjects e-cigarettes to many of the regulatory requirements of the Tobacco Control Act, including requiring warning labels on e-cigarette packaging and advertising. Our findings suggest text-only warnings for e-cigarettes may have limited impact on young adult non-smokers’ perceptions of e-cigarettes when they appear on e-cigarette ads. One goal of warnings for e-cigarette ads stated in the regulations is to inform consumers, particularly young non-tobacco users, about the potential harms and addictiveness of e-cigarettes [17, 18]. Our results indicate more effective strategies for designing e-cigarette warning label messaging may be needed to achieve this goal. Research on cigarette warning labels indicates factors such as the warning size, placement, message content, and features intended to draw visual attention affect consumer perceptions of tobacco products and related behavioral outcomes [35, 40–42]. With respect to warning message content, one recent experiment indicated warnings conveying that some e-cigarettes are produced by companies that make cigarettes may reduce craving to use cigarettes and e-cigarettes among young adult users of these products and reduce intentions to purchase e-cigarettes among young adult smokers and non-smokers [20]. Otherwise, there is currently limited evidence on whether such features of e-cigarette warnings could enhance their public health impact, and research in this area will be important to inform potential future e-cigarette regulatory requirements.

There has been speculation as to why e-cigarette manufacturers include warning labels on their advertisements prior to any regulatory requirements in the U.S., and before concrete evidence on the potential harms of long-term e-cigarette use is available. Manufacturers may do so to reduce their legal liability in the event products pose harms, and to attempt to develop a reputation of honesty and openness about the potential harms of their products [43]. However, the absence of regulations for e-cigarette advertisements until the recently published rule has meant that manufacturers have been free to test different warning messages to determine how they may affect their ads among consumers. Warnings used by manufacturers range widely, and this study attempted to capture the effects of different types of warnings used. A recent experimental investigation of the MarkTen warning label examined in this study showed that adult smokers and dual users of cigarettes and e-cigarettes notice the warning label, and those exposed to the warning were more likely to report that MarkTen e-cigarettes are harmful to health [21]. Our findings indicate the warnings used on ads for two major e-cigarette brands (MarkTen and Vuse), whose parent companies are also among the largest international cigarette companies (Altria, Inc., and Reynolds American, Inc., respectively), may have little effect among young adult non-smokers.

We also observed no significant differences across experimental conditions in behavioral intentions outcomes, which could be because a single brief exposure to e-cigarette ads and warning labels is insufficient [23] or due to limited interest in using these products among young non-smokers [3, 6]. Indeed, results of one recent study indicate that a small number of non-smokers included in the study indicated intentions to try e-cigarettes after they were exposed to a series of e-cigarette advertisements [44]. Our findings, along with the existing published research, indicate prospective studies with repeated exposures to e-cigarette warnings and that examine differences in their effects between young smokers and non-smokers will advance our understanding of these issues and inform potential future regulatory decisions.

The findings of this study should be interpreted in light of important limitations. All data are self-report and subject to potential reporting biases, and the online convenience sample limits generalizability of the findings. The study was conducted in a convenience sample recruited through an online data collection platform, limiting the potential generalizability of the findings to broader populations (e.g., those without internet access). We focused on young adult non-smokers based on the evidence that this is a population where e-cigarette use may occur, but future studies are needed to understand the impact of e-cigarette warning labels in other populations. Our study outcomes focused on perceptions and behavioral intentions and were assessed using primarily single item measures after a brief exposure to the experimental stimuli. This experimental approach, while informative, may not provide the ability to detect small changes in cognitive outcomes and prevents testing of potential mediation effects, such as whether changes in cognitive perceptions of e-cigarettes based on exposure to warning labels may impact behavioral outcomes. Additionally, in the experimental design we chose to match e-cigarette warnings with ads for a single e-cigarette brand (e.g., lengthy warning appeared only on MarkTen ads, FDA proposed warning appeared only on Blu ads). It is possible this may have affected our results, and in future experimental studies this could be addressed by randomizing different warnings of interest across brands. Also, participants randomized to the Ad Only and Ad with Warning conditions completed an additional heat mapping task, and it is unclear if this may have affected comparisons of outcomes between these two groups and the Warning Only condition. Finally, prior to the experimental exposure we measured lifetime e-cigarette use with a single, brief item and used this as a covariate in analyses. Use of more granular measures of prior and current e-cigarette use in future studies can help to discern whether the potential effects of e-cigarette ads and warning labels may differ based on the extent of previous e-cigarette use and/or current e-cigarette use.

## Conclusions

Despite these limitations, the results indicate the e-cigarette warning required by FDA and warning labels used by e-cigarette manufacturers are not likely to have much impact among young adult non-smokers when they appear on e-cigarette ads. Future tobacco regulatory research can build from this study by using prospective methods to examine potential changes in e-cigarette perceptions and behavioral intentions with repeated exposures to warning labels, broadening the focus to other populations of interest to FDA regulatory decisions (e.g., young adult smokers), and examining whether alternative strategies to designing e-cigarette warnings may enhance their impact. This line of investigation will be important to inform FDA tobacco regulatory decision-making since e-cigarettes are now subject to the requirements of the Tobacco Control Act, including required warning labels for e-cigarette advertisements.

## Abbreviations

E-cigarette, electronic cigarette; FDA, Food and Drug Administration

## Additional files

Additional file 1: E-cigarette advertising stimuli. (DOCX 5187 kb) Additional file 2: Results of heat-mapping task assessing visual attention to e-cigarette advertising stimuli. (DOCX 735 kb)
