# Supplementary material for: An experimental study of the effects of electronic cigarette warnings on young adult nonsmokers’ perceptions and behavioral intentions
Source: Tob Induc Dis. 2016 May 26;14:17. doi: 10.1186/s12971-016-0083-x (PMC4880975; doi:10.1186/s12971-016-0083-x)
Supplement: Additional file 1: — E-cigarette advertising stimuli. (DOCX 5187 kb) [file 12971_2016_83_MOESM1_ESM.docx]

**Additional File 1**

**Note: All branding content from e-cigarette ad images is visually occluded for publication purposes.**

**E-Cigarette Experimental Stimuli**

**
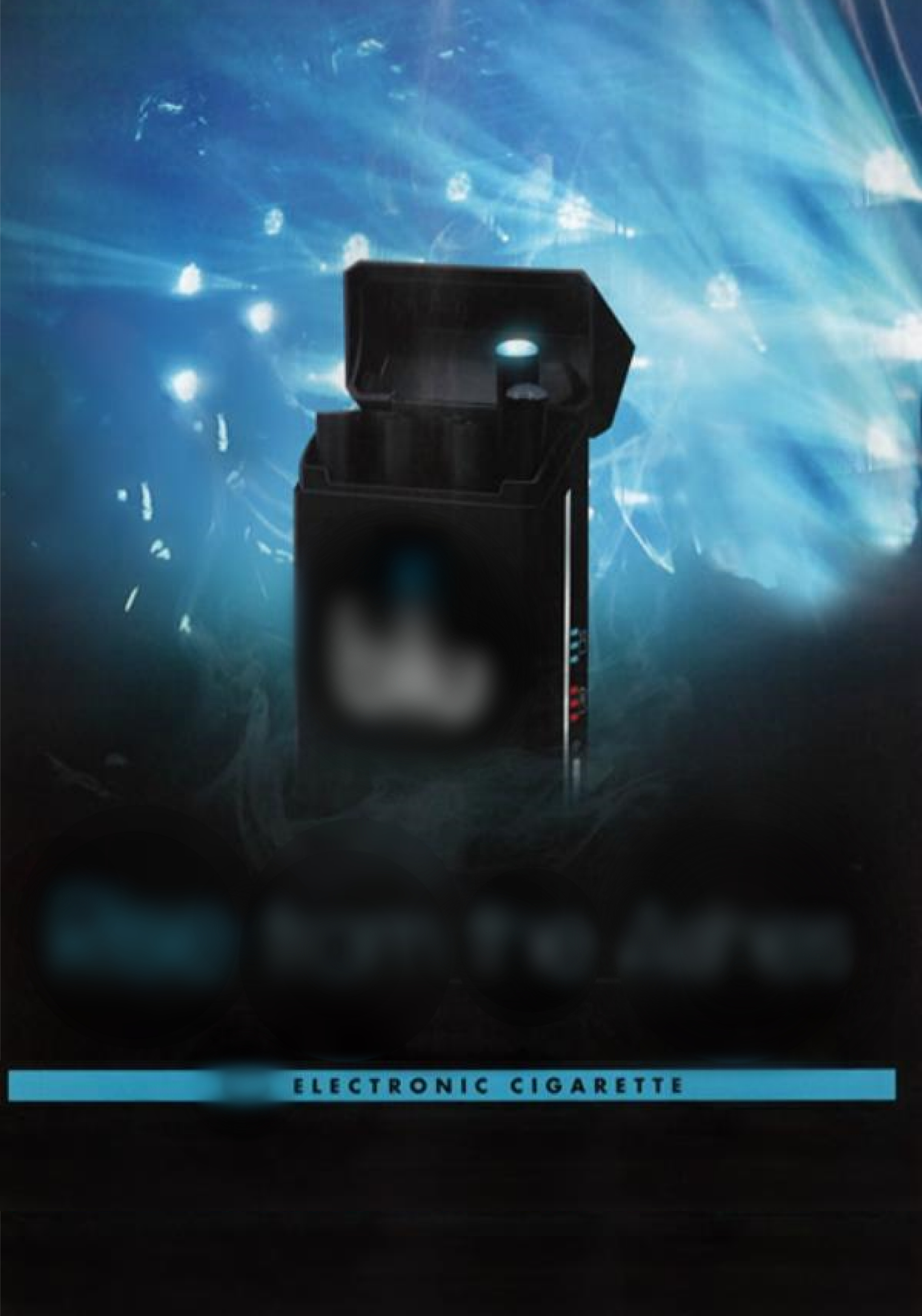

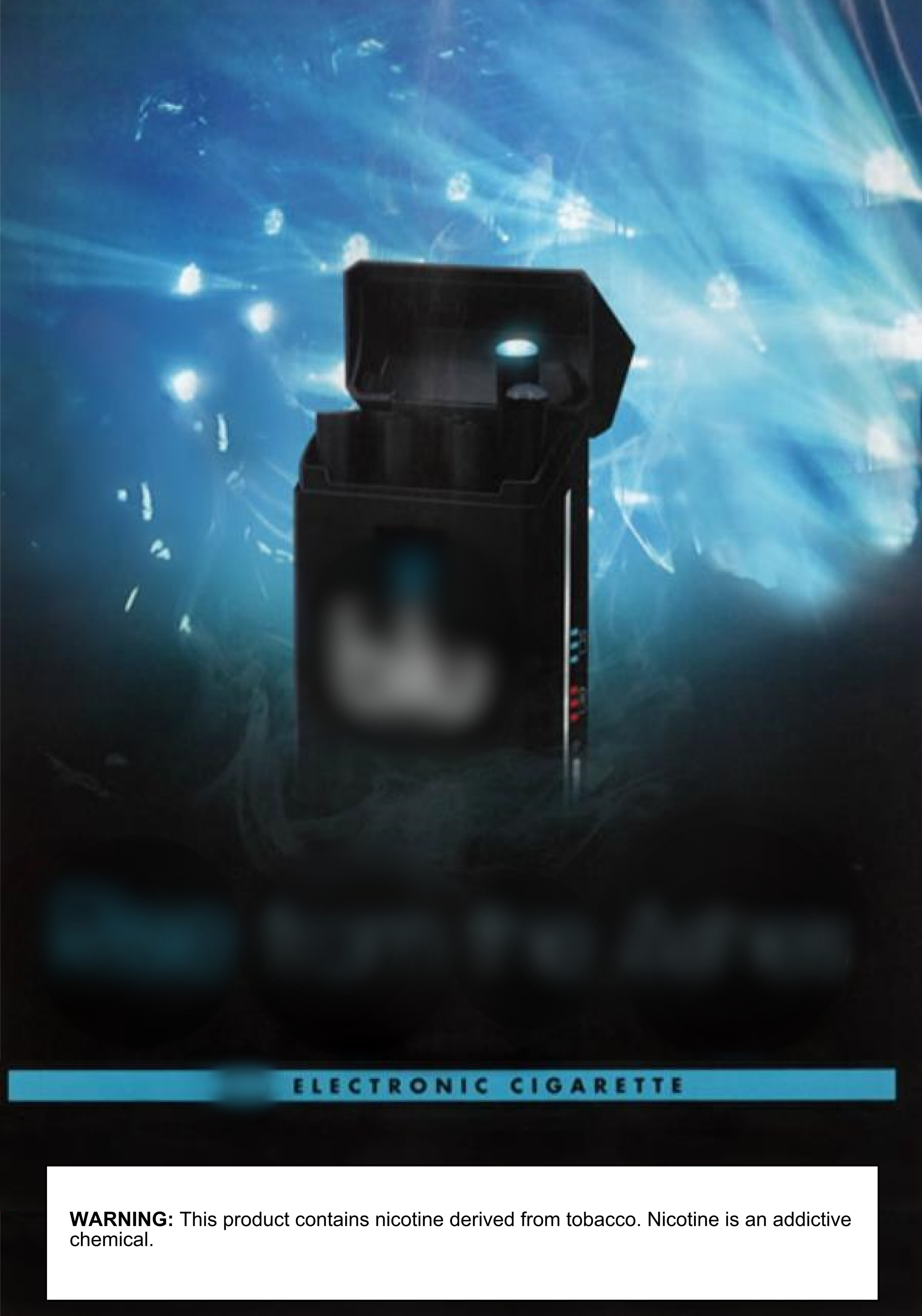

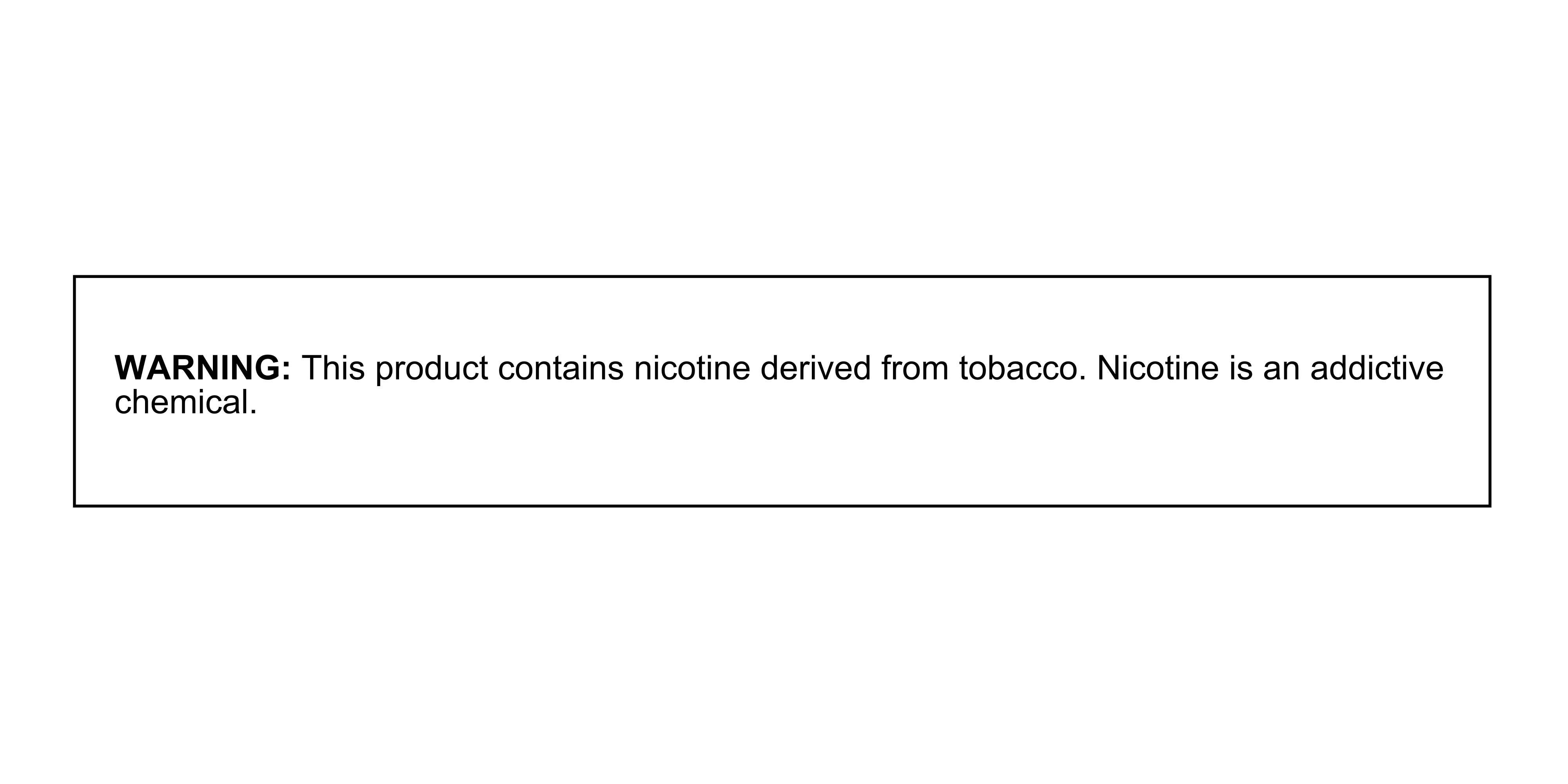
**

**Ad Only, Blu Ad with Warning, Blu Warning Only, Blu**

*Ad images credit: Trinketsandtrash.org*

**
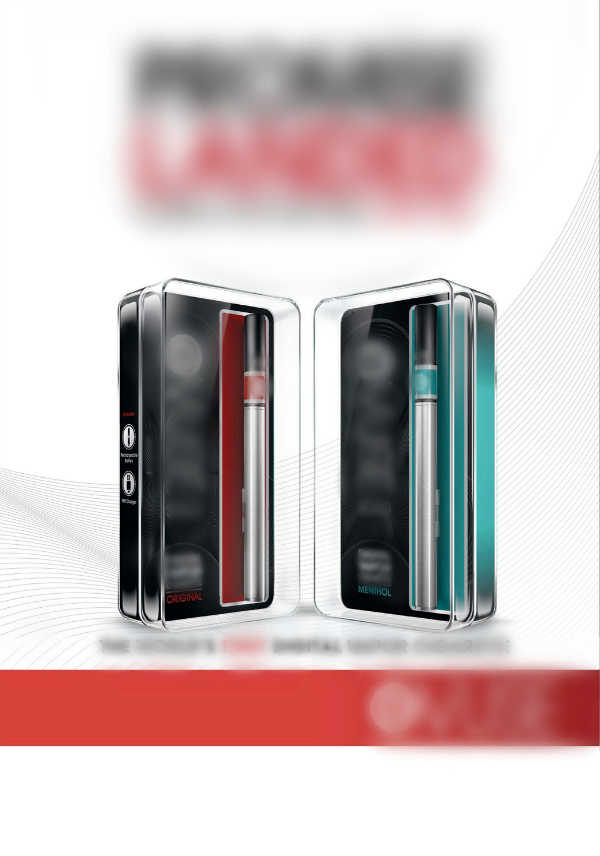

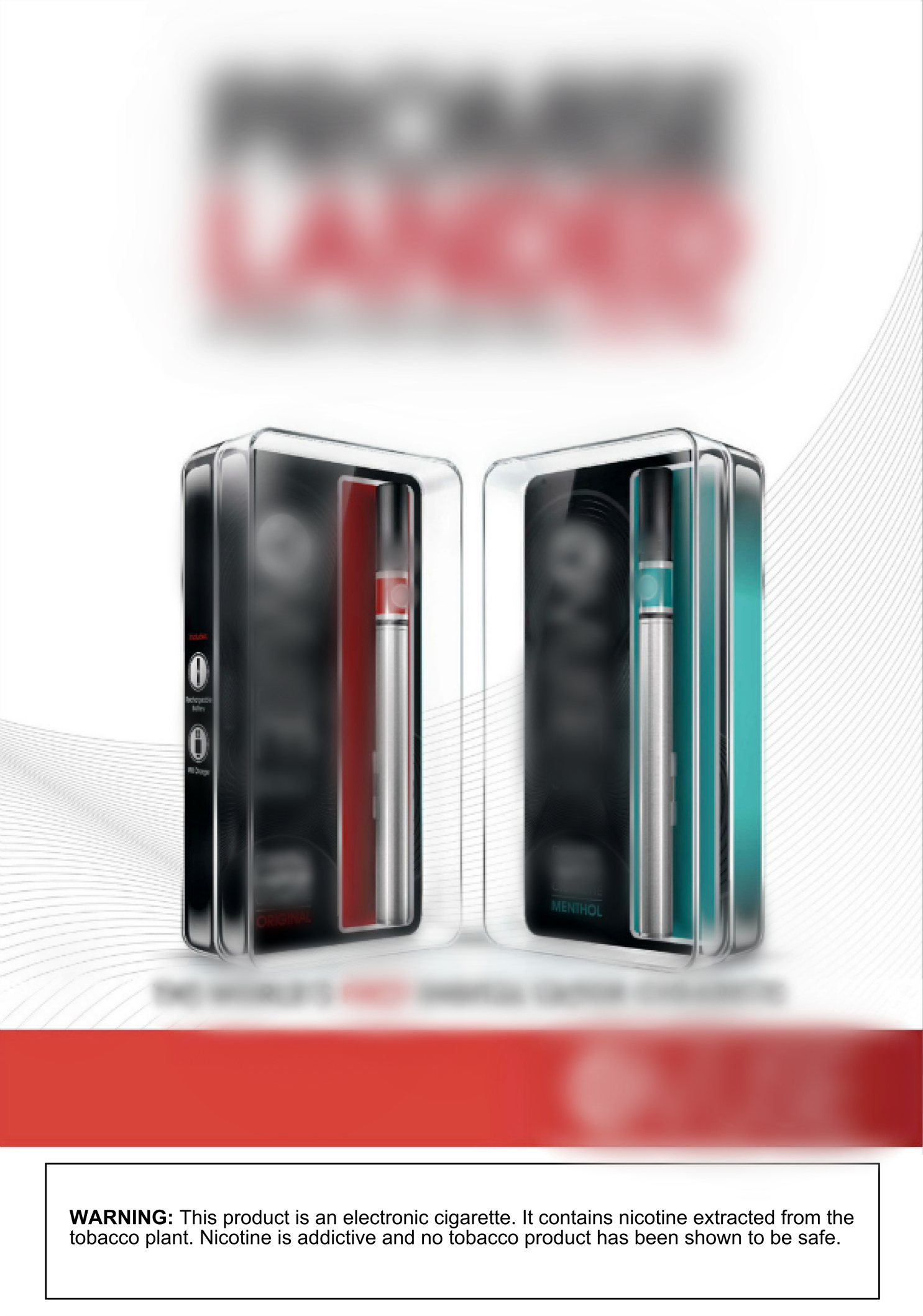

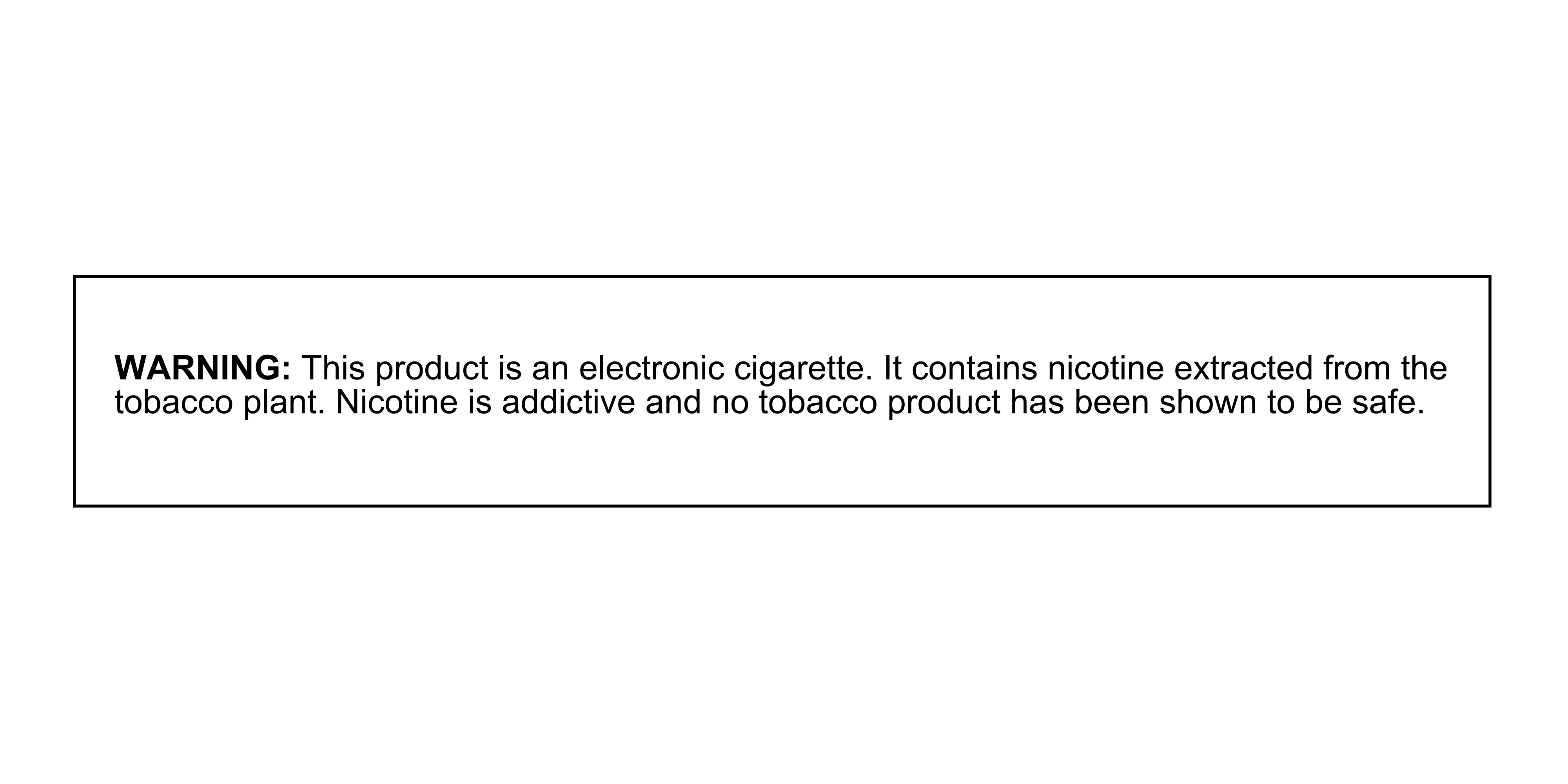
**

**Vuse, Ad Only Vuse, Ad with Warning Vuse, Warning Only**

*Ad images credit: Trinketsandtrash.org*

**
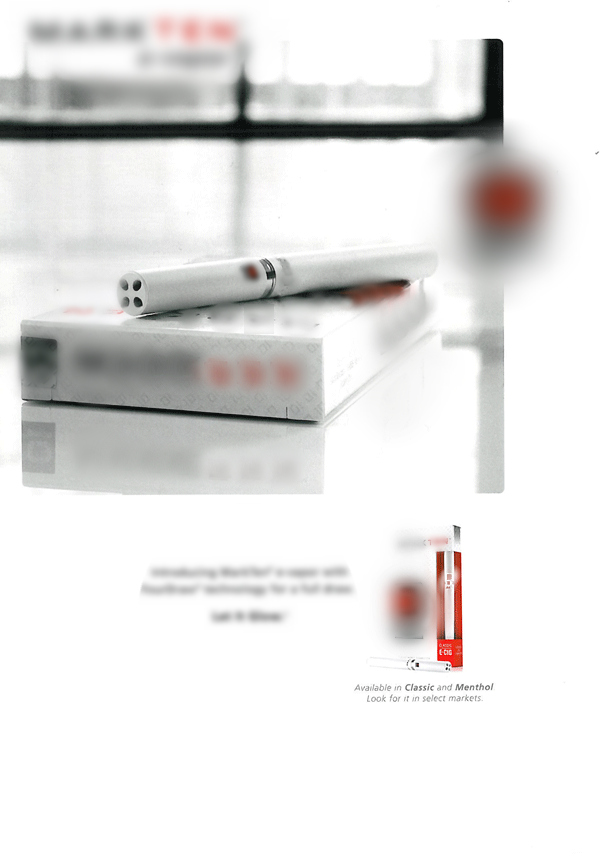

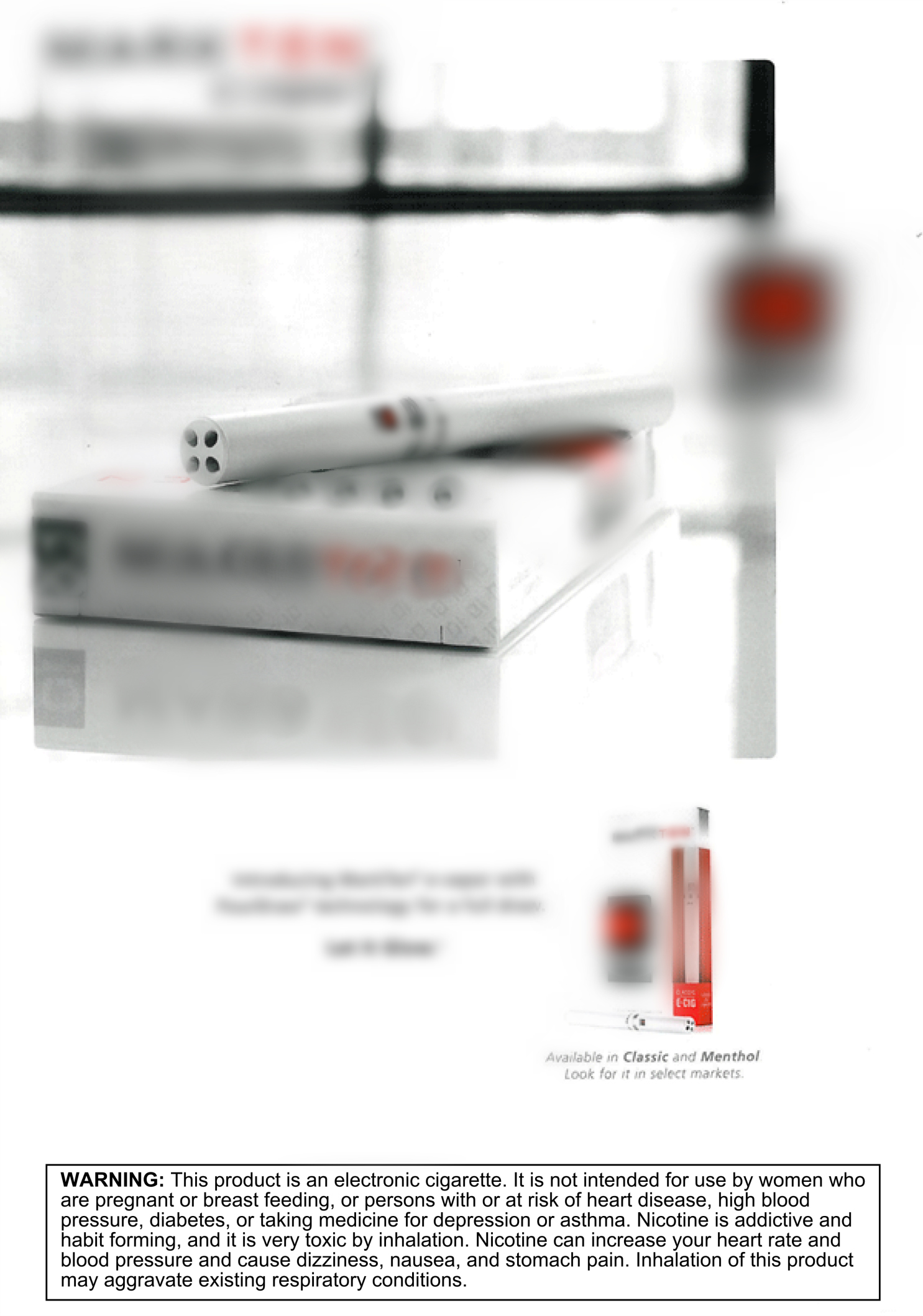

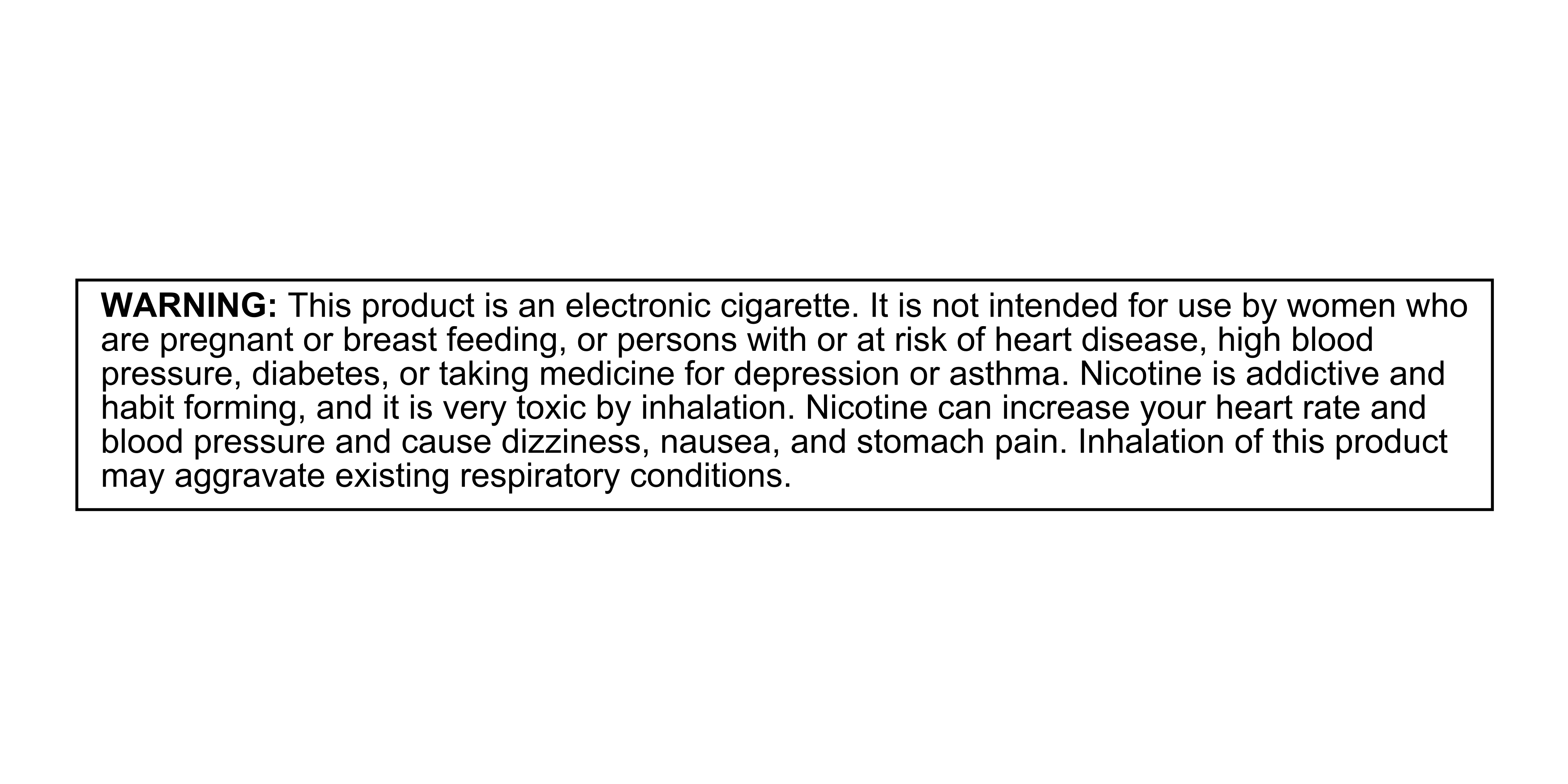
**

**MarkTen, Ad Only Mark Ten, Ad with Warning MarkTen, Warning Only***Ad images credit: Trinketsandtrash.org*
