# Supplementary material for: An experimental study of the effects of electronic cigarette warnings on young adult nonsmokers’ perceptions and behavioral intentions
Source: Tob Induc Dis. 2016 May 26;14:17. doi: 10.1186/s12971-016-0083-x (PMC4880975; doi:10.1186/s12971-016-0083-x)
Supplement: Additional file 2: — Results of heat-mapping task assessing visual attention to e-cigarette advertising stimuli. (DOCX 735 kb) [file 12971_2016_83_MOESM2_ESM.docx]

**Additional File 2**

**Note: All branding content from e-cigarette ad images is visually occluded for publication purposes.**

**Figure.** Heat map visual representation of areas of e-cigarette advertisements attracting participants’ attention


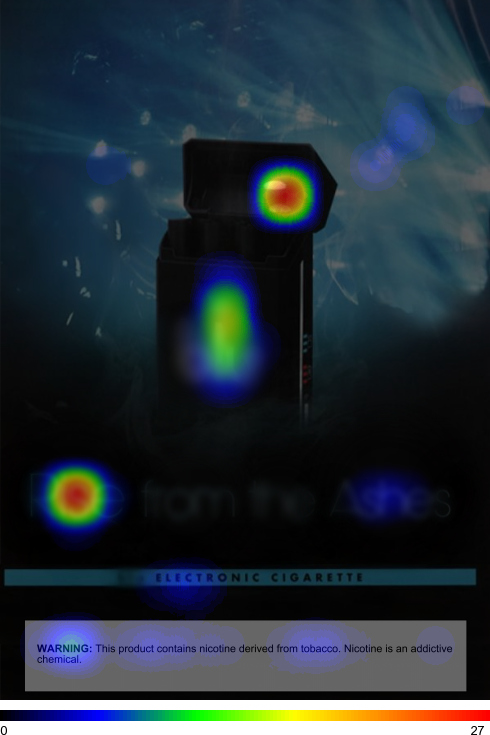

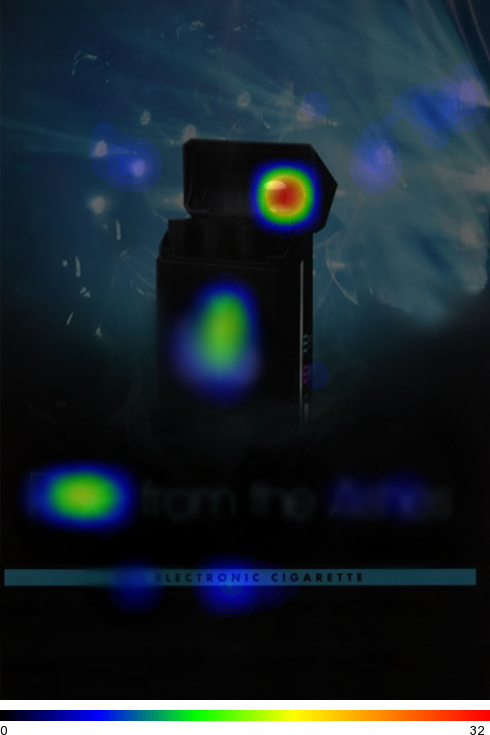

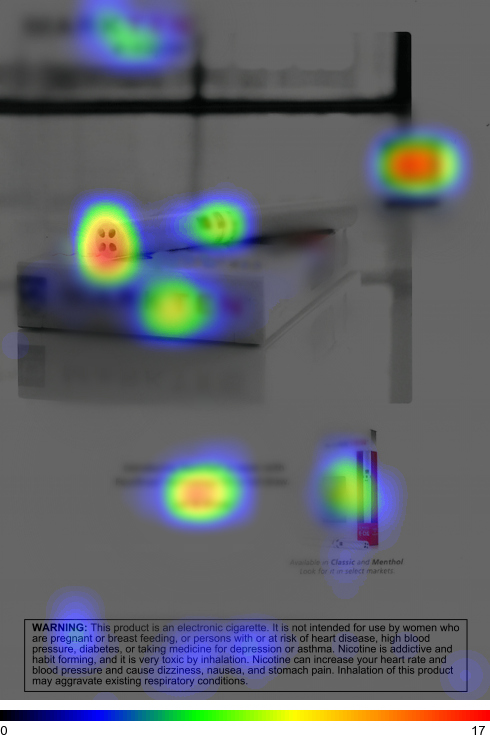

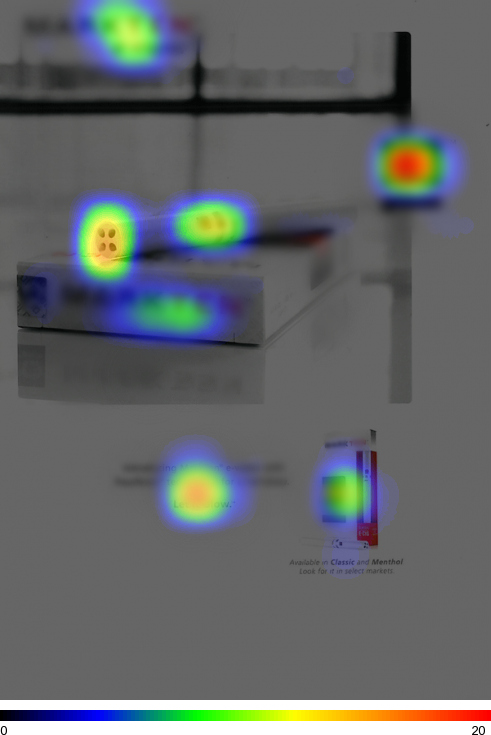

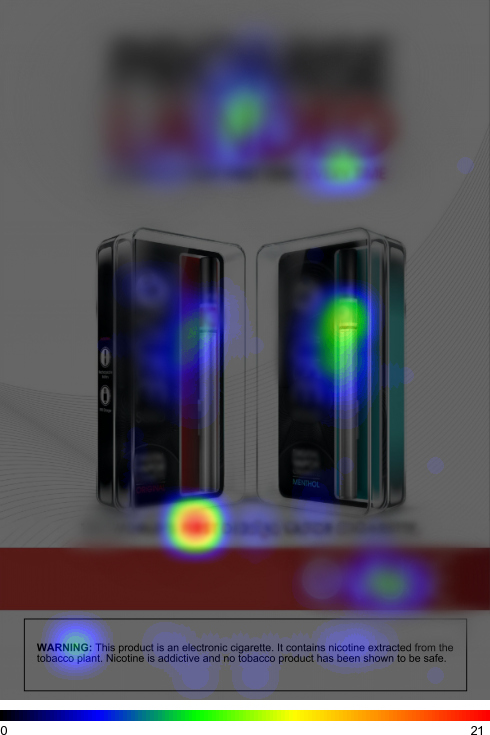

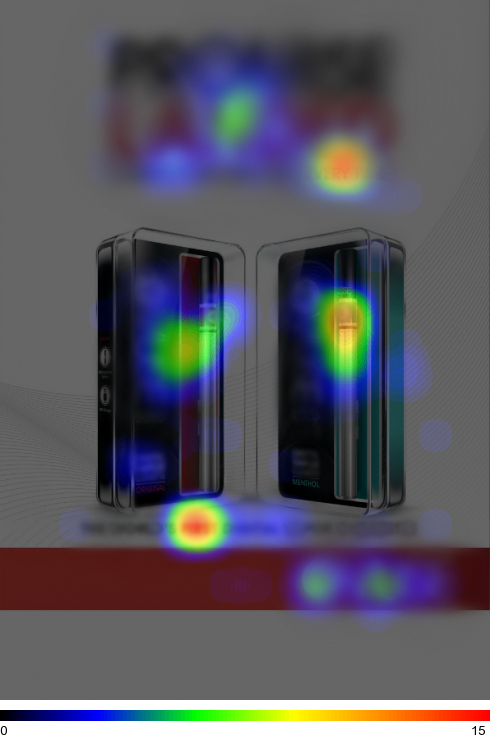


Note: For the heat map task participants in the Ad Only and Ad with Warning conditions were asked to select up to 5 areas of the ad that attracted their attention the most. Heat map colors indicate the proportion of participants reporting that a specific area of an ad attracted their attention. Coloring indicates the proportion of participants selecting an area of the ad, with higher proportions appearing in red and lower proportions appearing in blue. Ad images credit: Trinketsandtrash.org.
